# Factors Influencing Receipt of Iron Supplementation by Young Children and their Mothers in Rural India: Local and National Cross-Sectional Studies

**DOI:** 10.1186/1471-2458-11-617

**Published:** 2011-08-03

**Authors:** Sant-Rayn Pasricha, Beverley-Ann Biggs, NS Prashanth, H Sudarshan, Rob Moodie, Jim Black, Arun Shet

**Affiliations:** 1 The Nossal Institute for Global Health, Faculty of Medicine, Dentistry and Health Sciences, The University of Melbourne, Carlton, Victoria, Australia; 2 Department of Medicine, The Royal Melbourne Hospital, The University of Melbourne, Parkville, Victoria, Australia; 3 Hematology Research Unit, Division of Molecular Medicine, St. Johns National Academy of Health Sciences, Sarjapur Road, Bangalore 560034, India; 4 The Karuna Trust, B.R.Hills, Chamarajanagar, Karnataka, India; 5 Division of Global Health, Department of Public Health Sciences, Karolinska Institutet, Nobelsv 9, 171 77, Stockholm, Sweden

**Keywords:** Anaemia, Iron Deficiency, India, Children, Public Health

## Abstract

**Background:**

In India, 55% of women and 69.5% of preschool children are anaemic despite national policies recommending routine iron supplementation. Understanding factors associated with receipt of iron in the field could help optimise implementation of anaemia control policies. Thus, we undertook 1) a cross-sectional study to evaluate iron supplementation to children (and mothers) in rural Karnataka, India, and 2) an analysis of all-India rural data from the National Family Health Study 2005-6 (NFHS-3).

**Methods:**

All children aged 12-23 months and their mothers served by 6 of 8 randomly selected sub-centres managed by 2 rural Primary Health Centres of rural Karnataka were eligible for the Karnataka Study, conducted between August and October 2008. Socioeconomic and demographic data, access to health services and iron receipt were recorded. Secondly, NFHS-3 rural data were analysed. For both studies, logistic regression was used to evaluate factors associated with receipt of iron.

**Results:**

The Karnataka Study recruited 405 children and 377 of their mothers. 41.5% of children had received iron, and 11.5% received iron through the public system. By multiple logistic regression, factors associated with children's receipt of iron included: wealth (Odds Ratio (OR) 2.63 [95% CI 1.11, 6.24] for top vs bottom wealth quintile), male sex (OR 2.45 [1.47, 4.10]), mother receiving postnatal iron (OR 2.31 [1.25, 4.28]), mother having undergone antenatal blood test (OR 2.10 [1.09, 4.03]); Muslim religion (OR 0.02 [0.00, 0.27]), attendance at Anganwadi centre (OR 0.23 [0.11, 0.49]), fully vaccinated (OR 0.33 [0.15, 0.75]), or children of mothers with more antenatal health visits (8-9 visits OR 0.25 [0.11, 0.55]) were less likely to receive iron. Nationally, 3.7% of rural children were receiving iron; this was associated with wealth (OR 1.12 [1.02, 1.23] per quintile), maternal education (compared with no education: completed secondary education OR 2.15 [1.17, 3.97], maternal antenatal iron (2.24 [1.56, 3.22]), and child attending an Anganwadi (OR 1.47 [1.20, 1.80]).

**Conclusion:**

In rural India, public distribution of iron to children is inadequate and disparities exist. Measures to optimize receipt of government supplied iron to all children regardless of wealth and ethnic background could help alleviate anaemia in this population.

## Background

In India, approximately 55% of women of reproductive age and 69.5% of under-5 children are anaemic [1], and in children, over 70% of anaemia is attributable to iron deficiency [2]. Iron deficiency anaemia in mothers may be associated with an increased risk of maternal mortality, preterm delivery, and low birth weight [3]; and in children with reduced cognitive development [4]. Thus, anaemia is a major public health concern in India.

The Indian National Nutritional Anaemia Prophylaxis Programme recommends that all children aged 6 to 59 months, and all pregnant and lactating women, receive Iron Folic-Acid (IFA) [5]. Despite this policy, the prevalence of anaemia among toddlers aged 6-36 months has risen from 74.3% in 1998-9 to 78.9% in 2005-6 [1]. The third National Family Health Study (NFHS-3) examined receipt of iron and found that only 4.7% of children in India aged 6-59 months were receiving supplementation [1]. Several previous, local studies have also suggested that distribution of iron in India is poor [6,7].

An improved understanding of the coverage of and factors associated with receipt of iron supplements by mothers and children in India could inform efforts to strengthen and better direct iron delivery programmes, especially in rural India. We hypothesised that receipt of iron supplementation to children in India is poor, and is associated with socioeconomic conditions and access to health care. To test this hypothesis and better understand factors associated with the receipt of iron supplementation among children in rural India, we conducted two analyses. 1) In two districts of rural Karnataka (the 'Karnataka Study'), we undertook a cross-sectional study to evaluate the receipt of iron to young children and their mothers, and identify factors at the socioeconomic, demographic and health-care delivery levels that may contribute to disparities in receipt of supplementation. 2) We analysed data obtained from the third National Family Health Study (NFHS-3) performed across India, to evaluate factors associated with delivery of iron to children and their mothers throughout rural India.

## Methods

### The Karnataka Study

The study was performed between August and October 2008. A detailed description of the methodology of this study has been previously published [8].

#### Study site and participants

The study was based in two rural Primary Health Centres (PHCs) in southern Karnataka. The Gumballi PHC, in Chamarajnagar district, the southernmost district of Karnataka, is 180 km south of Bangalore, and provides care for about 21,700 people in 13 villages. The Sugganahalli PHC is 90 km west of Bangalore in the Ramnagara district, and provides care for 14,400 people in approximately 80 villages [9]. Agriculture comprises the major economic activity in both regions. The average rural income in Chamarajnagar in 2006 was (Indian rupees) Rs 22,006 per capita, in Ramnagara was Rs 26,009 [10], as compared with Karnataka overall Rs 26,123 [11]. In the 2011 Indian census, the Ramanagara district had a population of 1,082,739 and a literacy rate of 69.2%; Chamarajnagar had a population of 1,020,962 with a literacy rate of 61.1% [12].

The sampling design was developed to be acceptable to the local population and the Non-Government Organisation managing the PHCs. Three of four sub-centres managed by each PHC were randomly selected for involvement in the study [13]. All children aged 12 to 23 months living in the villages served by the selected sub-centres (enumerated by a compilation of local lists and a house to house survey), and their mothers, were eligible for inclusion in the study, unless children were unwell or febrile, or had ever received a previous blood transfusion (as this was part of a larger study investigating the determinants of anaemia in this population). Field workers visited each eligible village on a pre-publicised day and administered the questionnaire to and collected blood samples from all mothers of children living in the site who were eligible for recruitment. If the child presented with a guardian who was not their mother (for example, a grandmother, sibling or aunt), maternal blood samples and details of pregnancy were not obtained.

#### Study Procedures

The questionnaire evaluated demographics (religion and caste, age of mother and child, sex of child, education level and literacy of mother). A wealth index adapted from the NFHS-3 was also used, in which household assets are assigned a weighted score [14]. Subjects were allocated to wealth quintiles based on this score. Delivery of important maternal (antenatal and postnatal health worker visits) and child (vaccinations, vitamin A supplementation) primary health services were recorded, as was use of available resources including whether the child had ever visited an Anganwadi (Integrated Child Development Scheme) centre. Receipt of antenatal and postnatal iron supplementation by the mother was evaluated: mothers were shown bottles (liquid formulations) and strips (tablets) of iron supplements and asked to recall if they had received these. Mothers were also asked to recall whether their child had ever received iron, and if so, the source. The questionnaire is available online [8]. The field team was trained in administration of the questionnaire; all completed questionnaires were reviewed for errors, and cross-checked between interviewers.

Venous blood was collected from children for evaluation of haemoglobin (Sysmex XT-2000i, Sysmex Inc., Kobe, Japan) and capillary blood haemoglobin was estimated in their mothers by HemoCue (HemoCue 201+, Angelholm, Sweden). Anaemia in children was defined as haemoglobin < 11 g/dL, and in women as < 12 g/dL (11 g/dL if pregnant) [15].

### Third National Family Health Study

The NFHS-3 (performed in 2005-6 by the International Institute for Population Sciences (Mumbai, India) and ICF Macro (Washington DC, USA)) surveyed women aged 15-49 years and their children aged 0-59 months across all 29 Indian states. Respondents were selected through a multistage cluster-based survey stratified by urban and rural populations. Only rural families were included in our analysis. The rural sample was obtained through a selection of villages based on the probability proportional to size principle, followed by random selection of households [16]. The questionnaire recorded whether children were currently receiving iron and whether their mothers had received iron during their most recent pregnancy [17]. It also recorded child's age, sex, birth order, maternal education, family caste and religion, household wealth index, and health care practices.

### Statistical Considerations

Data from the Karnataka Study were entered into an EpiInfo database (EpiInfo 3.4.3, Centers for Disease Control and Prevention, Atlanta, USA) and exported to statistical software for analysis (Stata 11, StataCorp, College Station, Texas, USA). We calculated a sample size of 390 to ensure that the 95% confidence interval for an estimate of prevalence of 50% would have precision +/-5%. Significance for statistical tests was defined as p < 0.05. Associations with receipt of iron supplementation (a binary variable) were estimated using univariate and then multiple logistic regression. Wealth index and number of antenatal health worker visits were analysed as quintiles using dummy variables. The multiple regression models were fitted after forward stepwise logistic regression including all variables with retention of variables with p < 0.05. Each model was evaluated using likelihood ratio tests and confirmed using the Hosmer-Lemeshow goodness-of-fit test. T-tests were used to identify differences in continuous variables between groups.

NFHS-3 data was analysed with Stata 11 using the children's recode data (IAKR) file. Independent variables from the NFHS-3 questionnaire that reflected variables found to be associated with receipt of iron by mothers or children in the Karnataka Study were included in the analysis. Simple and multiple logistic regression models accounting for sample weights and the multistage cluster survey sampling design of the survey were used to identify associations with receipt of iron during pregnancy by the mother, or current receipt of iron by the child.

### Ethics

The Karnataka study was approved by ethics committees of St John's National Academy of Health Sciences, Bangalore, India, and the Faculty of Medicine, Dentistry and Health Sciences, the University of Melbourne, Australia. Written informed consent was obtained from mothers or guardians of all participating children. The NFHS-3 had received approval from institutional review boards of the International Institute of Population Sciences, Mumbai, India and the ORC Macro, Calverton, Maryland, USA. Verbal informed consent was obtained from participating mothers by the interviewers [17].

## Results

### The Karnataka Study

#### Demographics and delivery of health services

We estimated that 470 children were living in the selected villages and thus potentially eligible for the study; 415 (88.3%) presented for screening [8], of which 10 were excluded: 7 due to fever and 3 due to previous transfusion. Thus, 405 children were recruited. The sample included two pairs of twins; mother and child data was adjusted to avoid duplication. Of 403 included children, 203 (50.3%) were male. The profile of the sampled population of mothers and children is presented in Table 1. We found that 63.1% of mothers and 75.3% of the children were anaemic [2].

**Table 1:** Demographic parameters of mothers and children

	N	Mean [95% CI] Median [range] Prevalence n (% [95% CI])
**Mother**		
Age^1 ^(years)	376	23.2 [22.8, 23.6]
Years of education^2^	377	7 [0, 15]
Literate^3^	374	260 (69.5 [64.8, 74.2])
Wealth Index^1^	403	17.9 [16.7, 18.9]
Currently Pregnant^3^	374	45 (12.0 [8.7, 15.3])
Anaemic^3,4^	358	225 (62.8 [57.8, 67.9]
**Child**		
Age^1 ^(months)	401	17.2 [16.8, 17.5]
Male^3^	403	203 (50.4 [45.5, 55.3])
Birth order^2^	377	2 [1, 5]
Hindu, non Scheduled Caste^3^	390	209 (53.6 [48.6, 58.6])
Hindu, Scheduled Caste^3^	390	99 (25.4 [21.0, 29.7])
Scheduled Tribe^3^	390	61 (15.6 [12.0, 19.0])
Muslim^3^	390	21 (5.4 [3.1, 7.6])
Anaemic^3,5^	399	300 (75.2 [70.9, 79.4])

^1 ^Mean [95% CI]

^2 ^Median [range]

^3 ^Prevalence n (% [95% CI])

^4 ^Haemoglobin < 12 g/dL (non pregnant), <11 g/dL (pregnant)

^5 ^Haemoglobin < 11 g/dL

#### Receipt of iron supplements

Delivery of health services to and receipt of iron by mothers and children is presented in Table 2. Less than half of the children, 167/402 (41.5% [36.7-46.4]) had ever received iron supplements. Only 45/393 (11.5% [8.3-14.6]) had ever received iron from a government source (PHC, sub-centre or Anganwadi centre). The majority - 113/158 (71.5% [64.4-78.6]) - of children who had received iron received it from a private rather than a government source. The wealth index of families of those children who received iron from private sources was higher than those who received it from a government source (21.1 points [19.1-23.1] versus 17.0 points [14.0-20.7] p = 0.034). Most of the mothers (92.0% [89.2-94.8]) reported receipt of iron supplements during pregnancy, although the majority (80.8%) received 40 or fewer tablets.

**Table 2:** Delivery of health services and anaemia prophylaxis to mothers and their children

	N	n (% [95% CI])
**Health Services Provided to Mother**		
Antenatal visits by health worker^1^	375	374 (99.7 [99.2, 100.0])
Had postnatal health worker visit	370	167 ([45.1 (40.4, 50.3])
**Health Services Provided to Child**		
Vaccinations:		
Birth	403	394 (97.8 [96.3, 99.2])
6 weeks	399	386 (96.7 [95.0, 98.5 ])
10 weeks	399	384 (96.2 [94.4, 98.1])
14 weeks	399	366 (91.7 [89.0, 94.4])
9 months	403	393 (97.5 [96.0, 99.0])
Fully vaccinated	403	359 (89.1 [86.0, 92.1])
Vitamin A supplementation received ever	403	403 (100.0%)
Child has ever visited:		
Doctor	403	386 [95.8 (93.8, 97.8)]
Primary Health Centre	402	329 (81.8 [78.1, 85.6])
Anganwadi Centre	403	435 (85.6 [82.2, 89.1])
**Maternal anaemia prophylaxis measures**		
Blood test during pregnancy	374	292 (78.1 [73.9, 82.3])
Iron supplementation during pregnancy	376	345 (91.7 [89.0, 94.6])
Dose of iron received		
0 tablets	375	30 (8.0 [5.2, 10.7))
10-20 tablets	375	108 (28.8 [24.2, 33.4])
30-40 tablets	375	165 (44.0 [39.0, 49.1])
50-60 tablets	375	58 (15.5 [11.8, 19.1])
70-80 tablets	375	12 (3.2 [1.4, 5.0])
> 80 tablets	375	2 (0.5 [0.0, 1.3])
Iron supplementation post pregnancy	370	86 (23.2 [18.9, 27.6])
**Children's anaemia prophylaxis measures**		
Child had received iron supplementation	402	167 (41.5 [36.7, 46.4])
Source of iron supplementation		
Private doctor	161	91 (56.5 [48.8, 64.3])
Private shop	161	24 (14.9 [9.3, 20.5])
Primary Health Centre	161	35 (21.7 [15.3, 28.2])
Sub-centre health worker	161	8 (5.0 [1.6, 8.4])
Anganwadi worker	161	3 (1.9 [0.0, 4.0])

^1 ^Median number of antenatal health worker visits 6 [range 0, 9]

#### Factors associated with receipt of iron supplements

Results of simple logistic regression analyses for factors associated with receipt of antenatal iron supplements by mothers are presented in Table 3 and results of multiple logistic regression analyses of these associations are shown in Table 4. Results of simple logistic regression analyses for factors associated with receipt of iron by children are presented in Table 5 and results of multiple logistic regression analyses of these associations are shown in Table 6.

**Table 3:** Factors associated with mother receiving iron supplementation during pregnancy (univariate logistic regression)

Factor	Total Population		Iron supplements received (345/375)		Iron supplements not received (30/375)			p
	N	Prevalence n (% [95% CI]), Mean [95% CI], or Median [Range]	N	Prevalence n (% [95% CI]), Mean [95% CI], or Median [Range]	N	Prevalence n (% [95% CI]), Mean [95% CI], or Median [Range]	Odds Ratio [95% CI] for receipt of iron	
**Wealth quintile^1, 4^**								
**Quintile 1**	375	69 (18.4 [14.4, 22.4])	69	56 (81.2 [71.7, 90.6])	69	13 (18.8 [71.7, 90.6])	1.00 (Reference)	
**Quintile 2**	375	66 (17.6 [13.7, 21.4])	66	61 (92.4 [85.9, 99.0])	66	5 (7.6 [1.0, 14.1])	2.83 [0.95, 8.45]	0.062
**Quintile 3**	375	86 (22.9 [18.7, 27.2])	86	80 (93.0 [87.5, 98.5])	86	6 (7.0 [1.5, 12.4])	2.65 [1.0, 7.07]	0.051
**Quintile 4**	375	75 (20.0 [15.9, 24.1])	75	75 (93.3 [87.6, 99.1])	75	5 (6.7 [0.9, 12.4])	3.25 [1.09, 9.66]	0.034
**Quintile 5**	375	79 (21.1 [16.9, 25.2])	79	78 (98.7 [96.2, 100.0])	79	1 (1.3 [0.0, 3.7])	18.57 [2.36, 146.06]	0.005
**Mother's age (years)^2^**	374	23.2 [22.8, 23.6]	342	23.2 [22.8, 23.5]	31	23.9 [22.0, 25.8]	0.95 per year [0.87, 1.04]	0.275
**Child's age (months)^2^**	401	17.2 [16.8, 17.5]	343	17.0 [16.7, 17.4]	31	17.8 [16.3, 19.2]	0.94 per month [0.84, 1.04]	0.251
**Maternal Education^1^**								
**0**	375	89 (23.7 [19.4, 28.1])	89	80 (89.9 [83.5, 96.3])	89	9 (10.1 [3.7, 16.5])	1.00 (Reference)	
**1-6 years**	375	46 (12.3 [8.9, 15.6])	46	43 (93.5 [86.1, 100.0])	46	3 (6.5 [0, 13.9])	1.61 [0.42, 6.27]	0.491
**7-9 years**	375	117 (31.2 [26.5, 25.9])	117	107 (91.5 [86.3, 96.6])	107	10 (8.5 [3.4, 13.7])	1.20 [0.47, 3.10]	0.701
**10 years**	375	97 (25.9 [21.4, 30.3])	97	90 (92.8 [87.5, 98.0])	97	7 (7.2 [2.0, 12.5])	1.446 [0.52, 4.06]	0.484
**11+ years**	375	26 (6.9 [4.4, 9.5])	26	25 (96.2 [88.2, 100.0])	26	1 (3.8 [0, 11.8])	2.7 [0.33, 22.39]	0.358
**Literate mother^1^**	373	259 (69.4 [64.7, 74.1])	259	241 (93.1 [89.9, 96.2])	259	18 (6.9 [3.8,10.1])	1.58 [0.73, 3.39]	0.245
**Birth order^3^**	377	2 [1,5]	344	2 [1,5]	31	1 [1,4]	0.90 [0.58, 1.41]	0.634
**Religion/Caste^1^**								
**Hindu, non SC/ST^5^**	374	198 (52.9 [47.9, 58.0])	198	190 (96.0 [93.2, 98.7])	198	8 (4.0 [1.3, 6.8])	1.00 (Reference)	
**Scheduled Caste**	374	97 (25.9 [21.5, 30.4])	97	85 (87.6 [81.0, 94.3])	97	12 (12.4 [5.7, 19.0])	0.30 [0.12, 0.76]	0.011
**Scheduled Tribe**	374	58 (15.6 [11.8, 19.2])	58	52 (89.7 [81.6, 97.7])	58	6 (10.3 [2.3, 18.4])	0.7 [0.12, 1.09]	0.073
**Muslim**	374	21 (5.6 [3.3, 8.0])	21	17 (81.0 [62.6, 99.3])	21	4 (19.1 [0.7, 37.4])	0.18 [0.05, 0.66]	0.009
**Number of antenatal health worker visits^1,6^**								
**Quintile 1**	374	69 (18.5 [14.5, 22.4])	69	57 (82.6 [73.4, 91.8])	69	12 (17.3 [8.2, 26.6])	Reference	
**Quintile 2**	374	53 (14.2 [10.6, 17.7])	53	46 (86.8 [77.4, 96.2])	53	7 (13.2 [3.8, 22.6])	1.38 [0.50, 3.80]	0.529
**Quintile 3**	374	40 (10.7 [7.6, 13.8])	40	39 (97.5 [92.4, 100.0])	40	1 (2.5 [0.0, 7.6])	8.21 [1.02, 65.74]	0.047
**Quintile 4**	374	118 (31.6 [26.8, 36.3])	118	109 (92.3 [87.5, 97.2])	118	9 (7.6 [2.8, 12.5])	2.55 [1.01, 6.41]	0.047
**Quintile 5**	374	94 (25.1 [20.7, 29.6])	94	93 (98.9 [96.8, 100.0])	94	1 (1.1 [0.0, 3.2])	19.59 [2.48, 154.61]	0.005
**Postnatal health worker visit^1^**	369	167 (45.3 [40.2, 50.4])	167	164 (98.2 [96.2, 100.0])	167	3 (1.8 [0.0, 3.8])	8.43 [2.51, 28.33]	0.001
**Iron supplementation given post pregnancy^1^**	369	86 (23.3 [19.0, 27.6])	86	84 (97.7 [94.4, 100.0])	86	2 (2.3 [0.0, 5.6])	4.61 [1.08, 19.78]	0.040
**Blood test during pregnancy^1^**	373	291 (78.0 [73.8, 82.2])	291	276 (94.9 [92.3, 97.4])	291	15 (5.2 [2.6, 7.7])	4.12 [1.92, 8.84]	< 0.001

^1^Prevalence: n (% [95% CI])

^2^Mean [95% CI]

^3^Median [range]

^4^Wealth index: Quintile 1: 2-8 Quintile 2: 9-12; Quintile 3: 13-18; Quintile 4: 19-26; Quintile 27-62.

^5^SC = Scheduled Caste, ST = Scheduled Tribe

^6^Number of antenatal health worker visits: Quintile 1: 1-3; Quintile 2: 4; Quintile 3: 5; Quintile 4: 6-7; Quintile 8-9.

**Table 4:** Factors associated with mother receiving iron supplementation during pregnancy (multiple logistic regression)

Factor	Odds Ratio [95% CI]	P
**Postnatal health worker visit**	9.57 [2.50, 36.58]	0.001
**Wealth quintile^1^**		
**Quintile 1**	1.00 (Reference)	
**Quintile 2**	2.28 [0.61, 8.53]	0.223
**Quintile 3**	2.83 [0.84, 9.50]	0.093
**Quintile 4**	2.63 [0.71, 9.67]	0.146
**Quintile 5**	11.20 [1.17, 106.82]	0.036
**Religion/Caste**		
**Hindu, Non SC/ST^2^**	1.00 (Reference)	
**Scheduled Caste**	0.32 [0.11, 0.96]	0.041
**Scheduled Tribe**	0.63 [0.18, 2.19]	0.468
**Muslim**	0.11 [0.02, 0.56]	0.008
**Number of antenatal health worker visits^3^**		
**Quintile 1**	1.00 (Reference)	
**Quintile 2**	1.69 [0.51, 5.64]	0.394
**Quintile 3**	8.66 [0.93, 80.87]	0.058
**Quintile 4**	1.07 [0.35, 3.29]	0.908
**Quintile 5**	16.02 [1.83, 140.06]	0.012
**Blood test during pregnancy**	3.00 [1.19, 7.57]	0.020

^1^Wealth index: Quintile 1: 2-8 Quintile 2: 9-12; Quintile 3: 13-18; Quintile 4: 19-26; Quintile 27-62.

^2^SC = Scheduled Caste, ST = Scheduled Tribe

^3^Number of antenatal health worker visits: Quintile 1: 1-3; Quintile 2: 4; Quintile 3: 5; Quintile 4: 6-7; Quintile 8-9.

**Table 5:** Factors associated with child ever receiving iron supplementation (univariate logistic regression)

Factor	Total Population		Iron supplements received (345/375)		Iron supplements not received (30/375)			p
	N	Prevalence n (% [95% CI]), Mean [95% CI], or Median [Range]	N	Prevalence n (% [95% CI]), Mean [95% CI], or Median [Range]	N	Prevalence n (% [95% CI]), Mean [95% CI], or Median [Range]	Odds Ratio [95% CI] for receipt of iron	
**Wealth quintile^1, 4^**								
**Quintile 1**	402	77 (19.2 [15.3, 23.0])	77	24 (31.2 [20.6, 41.8])	77	53 (68.8 [58.3, 79.4])	Reference	
**Quintile 2**	402	71 (17.7 [13.9, 21.4])	71	25 (35.2 [23.8, 46.6])	71	46 (64.8 [53.4, 76.2])	1.20 [0.61, 2.38]	0.602
**Quintile 3**	402	91 (22.6 [18.5, 26.7])	91	35 (38.5 [28.3, 48.6])	91	56 (61.5 [51.4, 71.7])	1.38 [0.73, 2.62]	0.324
**Quintile 4**	402	79 (19.7 [15.8, 23.6])	79	37 (46.8 [35.6, 58.1])	79	42 (53.2 [41.9, 64.4])	1.95 [1.01, 3.74]	0.046
**Quintile 5**	402	84 (20.9 [16.9, 24.9])	64	46 (54.8 [43.9, 65.6])	84	38 (45.2 [34.4, 56.1])	2.67 [1.40, 5.10]	0.003
**Mother's age (years)^2^**	374	23.2 [22.8, 23.6]	161	23.1 [22.6, 23.6]	212	23.3 [22.8, 23.8]	0.98 per year [0.93, 1.04]	0.555
**Child's age (months)^2^**	401	17.2 [16.8, 17.5]	165	17.0 [16.5, 17.5]	235	17.3 [16.8, 17.7]	0.98 per month [0.92, 1.04]	0.445
**Maternal Education^1^**								
**0**	374	89 (23.8 [19.5, 28.1])	89	31.5 [21.6, 41.3]	89	68.6 [58.7, 78.4]	1.00 (Reference)	
**1-6 years**	374	45 (12.0 [8.7, 15.3])	45	53.3 [38.2, 68.5]	45	46.7 [31.5, 61.8]	2.49 [1.19, 5.20]	0.015
**7-9 years**	374	117 (31.3 [26.6, 36.0])	117	40.2 [31.2, 49.2]	117	59.8 [50.8, 68.8]	1.46 [0.82, 2.61]	0.199
**10 years**	374	98 (26.2 [21.7, 30.7])	98	48.0 [37.9, 58.0]	98	52.0 [42.0, 62.1]	2.01 [1.10, 3.65]	0.022
**11+ years**	374	25 (6.7 [4.1, 9.2])	25	60.0 [39.4, 80.6]	25	40.0 [19.4, 60.6]	3.27 [1.31, 8.17]	0.011
**Literate mother^1^**	373	259 (69.4 [64.7, 74.1])	259	48.3 [42.1, 54.4]	259	51.7 [45.6, 58.9]	2.02 [1.27, 3.21]	0.003
**Birth order^3^**	377	2 [1,5]	161	2 [1,5]	215	2 [1,5]	0.91 [0.70, 1.19]	0.495
**Religion/Caste^1^**								
**Hindu, non SC/ST^5^**	389	209 (53.7 [48.8, 58.7])	209	101 (48.3 [41.5, 55.2])	209	108 (51.7 [44.8, 58.5])	1.00 (Reference)	
**Scheduled Caste**	389	99 (25.4 [21.1 29.8])	99	34 (34.3 [24.8, 43.9])	99	65 (65.7 [56.1, 75.2])	0.56 [0.34, 0.92])	0.022
**Scheduled Tribe**	389	61 (15.7 [12.1, 19.3])	61	25 (41.0 [28.3, 53.6])	61	36 (59.0 [46.3, 71.7])	0.74 [0.42, 1.32])	0.313.
**Muslim**	389	20 (5.1 [2.9, 7.4])	20	1 (5.0 [0.0, 15.5])	20	19 (95.0 [84.5, 100.0])	0.06 [0.01, 0.43])	0.005
**Number of antenatal health worker visits ^1. 6^**								
**Quintile 1**	374	68 (18.2 [14.3, 22.1])	68	38 (55.9 [43.8, 68.0])	68	30 (44.1 [32.0, 56.2])	1.00 (Reference)	
**Quintile 2**	374	53 (14.2 [10.6, 17.7])	53	15 (28.3 [15.8, 40.8])	53	38 (71.7 [59.2, 84.2])	0.31 [0.15, 0.67]	0.003
**Quintile 3**	374	40 (10.7 [7.6, 13.8])	40	18 (45.0 [28.9, 61.1])	40	22 (55.0 [38.9, 61.1])	0.65 [0.29, 1.41])	0.276
**Quintile 4**	374	118 (31.6 [26.8, 36.3])	118	63 (53.4 [44.3, 62.5])	118	55 (46.7 [37.4, 55.7])	0.90 [0.60, 1.65])	0.742
**Quintile 5**	374	95 (25.4 [21.0, 29.8])	95	26 (27.4 [18.2, 36.5])	95	69 (72.6 [63.5, 81.8])	0.30 [0.15, 0.57]	< 0.0001
**Postnatal health worker visit^1^**	369	166 (45.0 [39.9, 50.1])	166	74 (44.6 [36.9, 52.2])	166	92 (55.4 [47.8, 63.1])	1.07 [0.71, 1.62]	0.740
**Iron supplementation given post pregnancy^1^**	369	86 (23.3 [19.0, 27.6])	86	84 (97.7 [94.4, 100.0])	86	2 (2.3 [0.0, 5.6])	4.61 [1.08, 19.78]	0.040
**Antenatal maternal iron supplementation^1^**	374	344 (92.0 [89.2, 94.7])	344	153 (44.5 [39.2, 49.8])	344	191 ((55.5 [50.3, 60.8])	2.20 [0.95, 5.08]	0.064
**Postnatal maternal iron supplementation^1^**	369	85 (23.0 [18.7, 27.4])	85	54 (63.5 [53.1, 73.9])	85	31 (36.5 [26.0, 46.9])	2.88 [1.74, 4.76])	< 0.0001
**Blood test during pregnancy^1^**	374	291 (78.0 [73.8, 82.2])	291	137 (47.1 [41.3, 52.8])	291	154 (52.9 [47.2, 58.7])	2.15 [1.27, 3.65]	0.005
**Child's sex (male = 1)^1^**	402	203 (50.5 [45.6, 55.4])	203	103 (50.7 [43.8, 57.7])	203	100 (49.3 [42.3, 56.2])	2.17 [1.45, 3.26]	< 0.001
**Services accessed ever**								
Doctor^1^	403	385 (95.7 [93.8, 97.5])	385	164 (42.6 [37.6, 47.6])	385	221 (57.4 [52.4, 62.4])	3.46 [0.98, 12.25]	0.054
Primary Health Centre^1^	402	328 (81.8 [78.0, 85.6])	328	138 (42.1 [36.7, 47.4])	328	190 (57.9 [52.6, 63.3])	1.10 [0.66, 1.84])	0.713
Anganwadi Centre^1^	402	345 (85.8 [82.4, 89.2])	345	124 (35.9 [30.8, 41.0])	345	221 (64.1 [59.0, 69.1])	0.18 [0.10, 0.35])	< 0.001
Child fully vaccinated^1^	402	359 (89.1 [86.0, 92.1])	358	136 (40.0 [32.9, 43.0])	358	222 (62.0 [57.0, 67.1])	0.6 [0.13, 0.51]	< 0.001

^1^Prevalence n (% [95% CI])

^2^Mean [95% CI]

^3^Median [range]

^4^Wealth index: Quintile 1: 2-8 Quintile 2: 9-12; Quintile 3: 13-18; Quintile 4: 19-26; Quintile 27-62.

^5^SC = Scheduled Caste, ST = Scheduled Tribe

^6^Number of antenatal health worker visits: Quintile 1: 1-3; Quintile 2: 4; Quintile 3: 5; Quintile 4: 6-7; Quintile 8-9.

**Table 6:** Factors associated with child ever receiving iron supplementation (multiple logistic regression)

Factor	Odds Ratio [95% CI]	P
**Child's sex (male = 1)**	2.45 [1.47, 4.10]	< 0.001
**Wealth quintile^1^**		
**Quintile 1**	1.00 (Reference)	
**Quintile 2**	0.89 [0.37, 2.16]	0.797
**Quintile 3**	1.31 [0.59, 2.90]	0.504
**Quintile 4**	1.65 [0.71, 3.83]	0.242
**Quintile 5**	2.63 [1.11, 6.24]	0.028
**Religion/Caste**		
**Hindu, non SC/ST^2^**	1.00 (Reference)	
**Scheduled Caste**	0.69 [0.38, 1.28]	0.240
**Scheduled Tribe**	0.90 [0.43, 1.77]	0.776
**Muslim**	0.02 [0.00, 0.27]	0.002
**Number of antenatal health worker visits^3^**		
**Quintile 1**	1.00 (Reference)	
**Quintile 2**	0.25 [0.10, 0.61]	0.003
**Quintile 3**	0.54 [0.21, 1.40]	0.201
**Quintile 4**	0.78 [0.37, 1.63]	0.506
**Quintile 5**	0.25 [0.11, 0.55]	< 0.001
**Visited Anganwadi**	0.23 [0.11, 0.49]	< 0.001
**Child fully vaccinated**	0.33 [0.15, 0.75]	0.008
**Postnatal maternal iron supplementation**	2.31 [1.25, 4.28]	0.008
**Maternal blood test during pregnancy**	2.10 [1.09, 4.03]	0.026

^1^Wealth index: Quintile 1: 2-8 Quintile 2: 9-12; Quintile 3: 13-18; Quintile 4: 19-26; Quintile 27-62.

^2^SC = Scheduled Caste, ST = Scheduled Tribe

^3^Number of antenatal health worker visits: Quintile 1: 1-3; Quintile 2: 4; Quintile 3: 5; Quintile 4: 6-7; Quintile 8-9.

No association was identified between anaemia in children and receipt of iron by either the child or their mother (for receipt of iron ever by children odds ratio (OR) 0.80 [0.51-1.27]; for receipt of any antenatal iron by mothers OR 1.97 [0.89-4.34]). Similarly, there was no association between anaemia in mothers and receipt of either antenatal or postnatal iron (receipt of any antenatal iron OR 1.10 [0.51-2.44], receipt of any postnatal iron OR 0.95 [0.57-1.60]).

### Third National Family Health Study

The dataset included 51,555 children and 36,850 of their mothers, of which 29,706 children and 20,631 mothers were coded as living in rural areas. Nationally, 4.6% [4.3-5.0] of all children were receiving iron at the time of interview: urban children were more likely to be receiving iron (7.0% [6.1-7.8]) than rural children (3.7% [3.3-4.1]) (OR for rural 0.55 [0.47-0.64], P < 0.001). Of mothers, 65.4% [64.0, 66.8] had received iron during their most recent pregnancy; women living in urban areas (73.9% [72.2-75.5]) were more likely to receive iron than rural mothers (61.1% [59.4-62.9]) (OR for rural 0.52 [0.50-0.55], P < 0.001). Because of differences between urban and rural India, further analysis was restricted to subjects living in rural India. Simple and multiple logistic regression analyses for factors associated with receipt of iron supplements by children and by mothers antenatally are presented in Table 7. The prevalence of each independent factor evaluated is presented in the NFHS-3 report [1].

**Table 7:** Factors associated with receipt of iron across rural India: National Family Health Study-3 data

	Antenatal iron supplements received by mother		Iron supplements currently received by children	
Factor	Odds Ratio [95% CI]	P	Odds Ratio [95% CI]	P
**Univariate logistic regression**				
Wealth quintile	1.51 [1.44, 1.57]	< 0.001	1.35 [1.26,1.45]	< 0.001
Maternal education				
0 years	Reference		Reference	
Primary	2.58 [2.30, 2.89]	< 0.001	1.76 [1.36, 2.28]	< 0.001
Secondary	4.11 [3.66, 4.61]	< 0.001	2.41 [1.96, 2.96]	< 0.001
Higher	14.00 [8.87, 22.08]	< 0.001	3.80 [2.61, 5.52]	< 0.001
Child's sex (male = 1)	0.98 [0.91, 1.05]	0.516	1.10 [0.95, 1.26]	0.191
Literate mother^1^	1.94 [1.84, 2.05]	< 0.001	1.47 [1.34, 1.61]	< 0.001
Birth order	0.78 [-.77, 0.80]	< 0.001	0.85 [0.81, 0.89]	< 0.001
Religion/Caste^2^				
Hindu, non SC/ST	1.21 [1.08, 1.34]	= 0.001	1.31 [1.09, 1.57]	= 0.005
Scheduled Caste	0.95 [0.85, 1.07]	= 0.418	0.71 [0.55, 0.92]	= 0.008
Scheduled Tribe	1.04 [0.88, 1.23]	= 0.657	1.06 [0.82, 1.38]	= 0.651
Muslim	0.74 [0.62, 0.87]	< 0.001	0.82 [0.61, 1.09]	= 0.167
Antenatal health worker visits (OR per visit)	1.77 [1.70, 1.85]	< 0.001	1.02 [1.01, 1.02]	< 0.001
Antenatal iron received by mother	N/A	N/A	3.02 [2.32, 3.93]	< 0.001
Postnatal iron received by mother	Not measured		Not measured	
Blood test during pregnancy	2.36 [2.10, 2.64]	< 0.001	2.50 [2.02, 3.10]	< 0.001
Services accessed:				
Anganwadi Centre^3^	2.03 [1.79, 2.30]	< 0.001	1.79 [1.47, 2.19]	< 0.001
Child vaccinated^4^	2.03 [1.73, 2.38]	< 0.001	1.28 [0.84, 1.94]	= 0.244
**Multiple logistic regression^5^**				
Wealth quintile	-		1.12 [1.02, 1.23]	= 0.019
Maternal education				
0 years	Reference		Reference	
Primary	1.60 [1.34, 1.91]	< 0.001	1.47 [1.02, 2.11]	= 0.039
Secondary	1.53 [1.31, 1.79]	< 0.001	2.15 [1.17, 3.97]	= 0.014
Higher	5.20 [2.65, 10.22]	< 0.001	3.17 [1.56, 6.44]	= 0.001
Literate mother^1^	-		0.74 [0.57, 0.97]	= 0.030
Birth Order	-		-	
Religion/Caste^2^				
Hindu, non SC/ST	-		-	
Scheduled Caste	-		-	
Scheduled Tribe	-		-	
Muslim	0.75 [0.62, 0.91]	= 0.003	-	
Antenatal health worker visits (OR per visit)	-		-	
Antenatal iron supplementation given to mother	N/A		2.24 [1.56, 3.22]	< 0.001
Blood test during pregnancy	2.06 [1.78, 2.38]	< 0.001	1.86 [1.47, 2.37]	< 0.001
Services accessed:				
Anganwadi Centre^3^	1.79 [1.51, 2.12]	< 0.001	1.47 [1.20, 1.80]	< 0.001
Child Vaccinated^4^	1.28 [1.06, 1.54]	= 0.010	-	

**^1^**Literacy defined as 0 = cannot read, 1 = can read part of a sentence, 2 = can read complete sentence; visually impaired mothers excluded.

^2^SC = Scheduled Caste; ST = Scheduled Tribe. Logistic regression performed for each category.

^3^Mother's use of Anganwadi in the last 3 months.

^4^Child ever had a vaccination

^5^Multiple regression coefficients only displayed for variables found to be significantly associated; variables not associated indicated with '-'.

## Discussion

The Indian Government has endorsed a policy, in line with World Health Organization recommendations [15], of providing routine iron supplementation to all children aged 6-60 months, and all pregnant and lactating women [5]. By conducting both a targeted study in two districts of rural Karnataka and also using data from a nationally representative survey, we have found a marked difference between anaemia control policy and iron supplement receipt in the field. We have also identified considerable disparities that are partly mediated by socio-economic and health care factors.

Private providers, rather than government health care workers, are the major source of supplements for children. Possible reasons for this may include, firstly, a lack of availability of appropriate supplements within the government system: inadequate supply of supplements in rural Indian PHCs has been previously observed [18], and government supplied liquid preparations suitable for children may be particularly difficult to access [19]. Iron preparations appropriate for young children are more expensive than iron tablets in retail pharmacies in India [20]. Secondly, government health workers and managers may be insufficiently educated about the importance of iron supplementation for prevention of anaemia in young children, and hence may not routinely provide this therapy [21]. Thirdly, wealthier families may be more likely to seek optimal health outcomes through private providers [22]. The unexpected finding in the Karnataka Study of an inverse association between children's receipt of iron supplementation and indicators of apparently good primary health care (receiving a full course of vaccinations and visits to the Anganwadi centre), together with the very low rate of distribution from government sources, raises the possibility that families who exclusively use government services are unable to receive iron supplements for their child through that system.

Data from both our field study and the NFHS-3 identify clear links between maternal anaemia control measures and delivery of iron to children. This may be due to improved awareness of anaemia and the value of iron in mothers, or to superior knowledge, attitudes and practices regarding anaemia prophylaxis in health workers caring for both mothers and their children. We have previously reported that children's haemoglobin concentrations in this population are chiefly associated with their iron status and their mother's haemoglobin [2]. Thus, controlling maternal anaemia may prevent anaemia in children as well as improve the health of the mother. Iron supplementation programmes for pregnant [23] and non-pregnant women [24] have been successfully implemented in several settings worldwide, including in India [25], and these programmes could be expanded with potential benefits for children as well as their mothers.

There are important differences between the methodology of the Karnataka Study and the NFHS-3. The two studies evaluated different outcomes: NFHS-3 asked whether children were *currently *receiving iron; the Karnataka Study asked whether children had *ever *received iron supplements. This may partly explain the lower national prevalence of receipt of iron identified on the NFHS-3: assuming all children received the nationally recommended 100 days of supplementation annually, and supplementation was evenly distributed across a year, 27% of children should have been receiving iron in the NFHS-3. However, the NFHS-3 data also indicated overall performance of services was poorer nationally than in the Karnataka Study. For example, whereas the Karnataka Study showed that 85.7% of children had ever visited an Anganwadi centre, NFHS-3 data shows that only 20.2% of children had visited a centre in the previous 3 months; this difference suggests heterogeneous access to ICDS services nationally that may explain the disparate findings in association between attendance at Anganwadi centres and receipt of iron between the two studies. Unlike the NFHS-3 data, the Karnataka study identified the source of iron received by children and thus was able to specifically evaluate government distribution. Although lower receipt of iron by children of Muslim families, as noted in the Karnataka Study, was not borne out nationally, this was identified among pregnant women from Muslim families in the national dataset. Finally, the Karnataka Study was prospectively designed with specific elements in the questionnaire to obtain a better understanding of the receipt of iron in rural India; the NFHS-3 dataset was used to perform a post-hoc analysis to understand iron receipt nationally.

Very few other published studies have reported receipt iron in the field by children in India. A study of 487 pregnant women in Andhra Pradesh identified receipt of iron by only 19%, and only 1% among children [7]. The Micronutrient Taskforce reviewed national nutritional anaemia control programmes in 1996 and identified limitations, including "poor compliance, irregular supplies, (and) low education/counseling" [26]. Thus, our study provides one of few comprehensive evaluations of iron supplementation both nationally, and in detail in a representative rural population.

The results of our study should be interpreted within the context of its strengths and limitations. This study analysed cross-sectional rather than longitudinal data, thus we are able to report only associations between variables, rather than definite cause and effect. Since the Karnataka Study sample size was relatively small, we sought to improve the external generalisability by also evaluating national (NFHS-3) data and making comparisons. Beyond variables measured in this study, there are likely to be multiple other factors that interact to affect the efficacy of anaemia control policies, concerning distribution and supply chains of iron supplements, performance of the health system as a whole, affordability of supplements, and acceptability of iron formulations to families.

Further research, including qualitative studies, are required to understand the gap between national anaemia control policy and practice in the field, and for disparities in receipt of iron supplements. Specifically, additional research is required to understand why receipt of iron was suboptimal in a setting where other vertical programmes (such as vaccination and Vitamin A distribution) function relatively well, as noted in the Karnataka Study. Such information may help to either specifically improve the iron supplementation programme or offer potential opportunities for synergy with these other programmes. Secondly, a study of the supply chain required for the provision of iron supplementation: from raw materials, manufacture, and distribution to the PHC, may help understand reasons for inadequate receipt of supplements that we did not address in this study. This could help programme managers plan for and procure sufficient stock of iron supplements for distribution through public systems. Thirdly, understanding the knowledge, attitudes and practices of government health workers and managers would help clarify how these factors affect implementation of anaemia control measures [27]. This information would help policymakers direct their management and training messages to improve iron supplementation through the government health system. Finally, research directed at understanding the likely acceptability of liquid iron formulations by children and their mothers in the field could be undertaken to address adherence to supplements, if or when they are made available.

Our finding, both locally and nationally, that children belonging to poorer families are less likely to receive iron (despite a higher burden of anaemia in poorer children [2]), is an example of the 'inverse care law': the poorest with greatest need have least access to valuable interventions [28]. Based on our results, improving the receipt of iron supplementation among all, but especially the poorest families, could potentially be achieved through education of health workers responsible for providing iron during pregnancy, in the post partum, and to children. Additionally, other primary health services offer an opportunity to introduce iron supplementation, for example, integrated delivery with 9 and 18 month vaccinations or with Vitamin A supplements [29]. Once women have experienced health benefits from iron, they may be more committed to continuing iron supplementation themselves [30] and may also be more likely to seek iron supplementation for their children, as suggested by our data. However, beyond these strategies, emphasis must also be given to further developing longer-term strategies to eliminate anaemia including; development of effective alternatives to iron supplementation, such as home fortification by microencapsulated micronutrients [31], iron fortification of staple foods, condiments and complementary foods [32], and dietary diversification.

## Conclusions

Despite an enormous and deteriorating problem of anaemia in India, iron supplementation policies for rural children and mothers are inadequately implemented. Key factors associated with access to iron supplements are wealth and maternal access to health services; ethnic disparities seem to be important, and improved maternal access to anaemia control measures also benefits access for children. Ensuring optimal delivery of iron supplements to all children and pregnant women, regardless of their socioeconomic background, could help address the enormous burden of anaemia in this population.

## Competing interests

The authors declare that they have no competing interests.

## Authors' contributions

SP, BA-B, JB, and AS planned the study and prepared the manuscript. SP and AS led the fieldwork. SP, BA-B and JB analysed the data. All authors conceived the study, read and approved the final manuscript.

## Pre-publication history

The pre-publication history for this paper can be accessed here:

http://www.biomedcentral.com/1471-2458/11/617/prepub

## References

[B1] IIPS National Family Health Survey (NFHS-3), 2005-06 2007 1 Mumbai: International Institute for Population Sciences (IIPS) and Macro International

[B2] Pasricha S-R Black J Muthayya S Shet A Bhat V Nagaraj S Prashanth NS Sudarshan H Biggs B-A Shet AS Determinants of Anemia Among Young Children in Rural India Pediatrics 2010 126 1 10.1542/peds.2009-3108 20547647

[B3] Allen LH Anemia and iron deficiency: effects on pregnancy outcome Am J Clin Nutr 2000 71 5 Suppl 1280S 1284S 10799402 10.1093/ajcn/71.5.1280s

[B4] Sachdev H Gera T Nestel P Effect of iron supplementation on mental and motor development in children: systematic review of randomised controlled trials Public Health Nutr 2005 8 2 117 132 15877905 10.1079/phn2004677

[B5] Saxena S Review of the policy regarding micronutrients - Iron Folic Acid (IFA) 2007 New Delhi: Ministry of Health and Family Welfare, Government of India

[B6] Malagi U Reddy M Naik RL Evaluation of National Nutritional Anaemia Control Programme in Dharwad (Karnataka) J Hum Ecol 2006 20 4 279 281

[B7] Vijayaraghavan K Brahmam GN Nair KM Akbar D Rao NP Evaluation of national nutritional anemia prophylaxis programme Indian J Pediatr 1990 57 2 183 190 10.1007/BF02722084 2246014

[B8] Pasricha SR Vijaykumar V Prashanth N Sudarshan H Biggs BA Black J Shet A A community based field research project investigating anaemia amongst young children living in rural Karnataka, India: a cross sectional study BMC Public Health 2009 9 1 59 10.1186/1471-2458-9-59 19222859 PMC2667415

[B9] PHC Monthly Data 2008 Bangalore: Karuna Trust

[B10] http://www.xe.com/ucc/convert.cgi

[B11] Bhandari L Kale S Karnataka: Performance, Facts and Figures 2007 Delhi: Indicus Analytics

[B12] Provisional Population Totals - Census 2011: Karnataka http://www.censusindia.gov.in/2011-prov-results/prov_data_products_karnatka.html

[B13] 1998 Dublin: School of Computer Science and Statistics, Trinity College http://random.org

[B14] Rutstein SO Johnson K The DHS Wealth Index 2004 Calverton, Maryland: ORC Macro

[B15] WHO/UNICEF/UNU Iron Deficiency Anaemia: Assessment, Prevention, and Control. A guide for programme managers 2001 Geneva: World Health Organization

[B16] Subramanian SV Ackerson LK Davey Smith G John NA Association of maternal height with child mortality, anthropometric failure, and anemia in India JAMA 2009 301 16 1691 1701 10.1001/jama.2009.548 19383960 PMC3095774

[B17] International Institute for Population Sciences (IIPS) and Macro International National Family Health Survey (NFHS-3), 2005-06: India 2007 2 Mumbai: IIPS

[B18] Chaturvedi S Ranadive B Are we really making motherhood safe? A study of provision of iron supplements and emergency obstetric care in rural Maharashtra Natl Med J India 2007 20 6 294 296 18335795

[B19] Kapil U Prevention and control of iron deficiency anemia amongst young children Indian Pediatr 2003 40 4 293 295 12736399

[B20] Rationale of Iron Dosage and Formulations in Under Three Children http://www.idpas.org/111c-india.html

[B21] Bhandari N Mazumder S Bahl R Martines J Black RE Bhan MK Use of multiple opportunities for improving feeding practices in under-twos within child health programmes Health Policy Plan 2005 20 5 328 336 10.1093/heapol/czi039 16113403

[B22] Das J Hammer J Location, location, location: residence, wealth, and the quality of medical care in Delhi, India Health Aff (Millwood) 2007 26 3 w338 351 10.1377/hlthaff.26.3.w338 17389631

[B23] Pena-Rosas JP Nesheim MC Garcia-Casal MN Crompton DW Sanjur D Viteri FE Frongillo EA Lorenzana P Intermittent iron supplementation regimens are able to maintain safe maternal hemoglobin concentrations during pregnancy in Venezuela J Nutr 2004 134 5 1099 1104 15113952 10.1093/jn/134.5.1099

[B24] Casey GJ Phuc TQ Macgregor L Montresor A Mihrshahi S Thach TD Tien NT Biggs BA A free weekly iron-folic acid supplementation and regular deworming program is associated with improved hemoglobin and iron status indicators in Vietnamese women BMC Public Health 2009 9 261 10.1186/1471-2458-9-261 19630954 PMC2720967

[B25] Vir SC Singh N Nigam AK Jain R Weekly iron and folic acid supplementation with counseling reduces anemia in adolescent girls: a large-scale effectiveness study in Uttar Pradesh, India Food Nutr Bull 2008 29 3 186 194 18947031 10.1177/156482650802900304

[B26] Kapur D Agarwal KN Agarwal DK Nutritional anemia and its control Indian J Pediatr 2002 69 7 607 616 10.1007/BF02722690 12173702

[B27] Aikawa R Jimba M Nguen KC Zhao Y Binns CW Lee MK Why do adult women in Vietnam take iron tablets? BMC Public Health 2006 6 1 144 10.1186/1471-2458-6-144 16753068 PMC1526427

[B28] Hart JT The inverse care law Lancet 1971 1 7696 405 412 4100731 10.1016/s0140-6736(71)92410-x

[B29] Kapil US Technical consultation on Strategies for Prevention and Control of Iron Deficiency Anemia amongst under three children in India Indian Pediatr 2002 39 7 640 647 12147889

[B30] Casey GJ Jolley D Phuc TQ Tinh TT Tho DH Montresor A Biggs BA Long-term weekly iron-folic acid and de-worming is associated with stabilised haemoglobin and increasing iron stores in non-pregnant women in Vietnam PLoS One 2010 5 12 e15691 10.1371/journal.pone.0015691 21209902 PMC3012714

[B31] Zlotkin S Control of anemia: the time to act is now Indian Pediatr 2007 44 2 84 86 17351298

[B32] Allen L de Benoist B Dary O Hurrell R Guidelines on food fortification with micronutrients Geneva: WHO 2006

